# Effects of Land Use Changes from Paddy Fields on Soil Bacterial Communities in a Hilly and Mountainous Area

**DOI:** 10.1264/jsme2.ME15187

**Published:** 2016-04-19

**Authors:** Md Rokunuzzaman, Yumiko Ueda, Li Chen, Sota Tanaka, Kouhei Ohnishi

**Affiliations:** 1 The United Graduate School of Agricultural Sciences, Ehime University3–5–7 Tarumi, Matsuyama, Ehime 790–8566Japan; 2 Faculty of Agriculture, Kochi University200 Monobe, Nankoku, Kochi 783–8502Japan; 3 College of Food Engineering and Nutritional Science, Shaanxi Normal UniversityXi’an, Shaanxi 710062China; 4 Graduate School of Kuroshio Science, Kochi University200 Monobe, Nankoku, Kochi 783–8502Japan; 5 Research Institute of Molecular Genetics, Kochi University200 Monobe, Nankoku, Kochi 783–8502Japan

**Keywords:** DGGE, pyrosequencing, land use, paddy field

## Abstract

Soil bacterial community structures in terraced rice fields and abandoned lands in a hilly and mountainous area were analyzed using 16S rRNA gene sequences. The DGGE band patterns of each soil were similar. Based on pyrosequencing data, the richness and diversity of bacterial species were slightly higher in paddy fields than in other soils. A beta-diversity analysis clearly indicated that the bacterial community structure in paddy fields differed from those in non-paddy field lands and crop fields that had not been used as a paddy field. These results may reflect the history of land use.

Soils are regarded as dynamic living substances and the most diverse microbial habitats on Earth. Bacterial communities in soil are very important particularly for agriculture and vary from soil to soil depending on the environment. Several factors are known to affect bacterial community structures, such as soil physicochemical properties (1, 13, 15), pH (26), vegetation cover (15), and disturbances (5). Land use and agricultural management are major causes of losses in biodiversity with resultant negative consequences for the environment (2, 20). On the other hand, agricultural practices do not always deplete soil bacterial diversity because shifts in microbial diversity and structure caused by different land uses may have a positive, negative, or neutral impact (24). The taxonomic composition of soil bacterial communities may also be varied by land use; for example, vegetation types (15), the conversion of native vegetation to agriculture (21, 25), and agriculture management practices (3, 31), all of which correlate with changes in the taxonomic composition; however, these changes often co-vary with alterations in soil chemistry.

Agriculture in hilly and mountainous areas is very important in Kochi Prefecture, Japan, because more than 80% of this prefecture is covered by forest and plain land is limited. Although agriculture was widely practiced in mountainous terraced paddy fields more than 30 years ago, many paddy fields have since been abandoned and have remained unused for a long time. A large number of studies have been conducted on the isolation of soil microbes and evaluation of microbial communities for agricultural aspects in Japan (10, 16). However, few studies have examined soil bacterial populations in long-term abandoned land. In order to achieve the future reuse of abandoned land as paddy fields, it is important to evaluate the effects of land use changes from paddy fields on the soil bacterial community in hilly and mountainous areas. Therefore, the population composition and diversity of soil bacteria were herein investigated using DGGE and 454 pyrosequencing of 16S rRNA genes amplified from DNA extracted directly from collected soil samples. We performed DGGE in order to obtain an overview of differences and similarities in the bacterial community structures of these soil samples. Pyrosequencing was employed to precisely analyze bacterial community structures.

Soil sampling was conducted in Nuta Village, which is located on a middle slope with a gradient of approximately 20° in the mountainous area of Otoyo Town, Kochi, Japan (at lat. 33°47′10″ N and log. 133°47′07″ E) on March 29 and 30, 2013. Soil samples were collected from seven land use types: paddy fields (P), abandoned paddy fields (AL), Yuzu citrus gardens (*Citrus junos*, C), planted forests (PF), vegetable crop fields (CF), bamboo thickets (*Bambuseae* spp., B), and a ginkgo garden (*Ginkgo biloba.* G). Soil samples were collected from depths of 0–15 cm and 15–30 cm in triplicate, and were mixed well to make one composite sample that was air-dried and passed through a 2-mm mesh sieve for a general physicochemical analysis. Only soils from 0–15 cm were used in the DNA analysis. Na^+^, K^+^, Mg^2+^, and Ca^2+^ concentrations were determined using an ion analyzer (IA-300, DKKTOA, Tokyo, Japan). Available P was measured by Troug’s method (27). Total carbon and nitrogen contents were assessed using an NC analyzer (JM1000CN, J-Science, Kyoto, Japan). NH_4_^+^-N and NO_3_^−^-N concentrations were determined using the steam distillation method (17).

Twenty-five sampling sites were selected ranging from 381 to 655 m above sea level (asl.) on the slope (Table S1). These were composed of 6 terraced paddy fields before irrigation (P1 to P6), 6 abandoned terraced paddy fields (AL1 to AL6), 4 citrus gardens (C1 to C4), 3 planted forests (*Chamaecyparis obtusa* for PF1, *Cryptomeria japonica* for PF2 and PF3), 2 vegetable crop fields (CF1 and CF2), a ginkgo garden (G1), and 3 bamboo thickets (B1 to B3). The sites with similar altitudes were adjacent or at least located close to each other. AL were abandoned 10 to 30 years ago and had since been covered with herbaceous species (mainly *Poaceae* spp.). C1–4 and PF1 and PF2 were converted 7 to 8 years ago and more than 30 years ago, respectively. PF3, CF1 and CF2, and G1 were located on the natural slope.

In terms of differences in the altitudes of the sampling sites, no appreciable feature was observed in the properties of soils from 0–15 cm. The soil texture class was silty clay loam or finer because the parent materials were derived from the green rock. The cation exchange capacity (CEC) was high (Table S1). Although soils were acidic, CF1–2 and G1 were close to neutral pH (Table S1). Available P was higher in P1–6, C1–4, and CF1–2 (Table S1). Soils from 15–30 cm showed similar results (Table S2).

DNA was extracted from 0.5 g of soil using ISOIL for Beads Beating (Nippon Gene, Tokyo, Japan) in combination with a bead beater at 5,500 rpm for 45 s (Micro Smash MS-100, Tomy Seiko, Tokyo, Japan). The V3 region of the 16S rRNA gene was amplified by the universal primers, 341F-GC and 534R (Table S3), with NEB Taq polymerase (New England Biolabs, Ipswich, MA, USA). PCR products were purified by Agencourt AMPure XP (Beckman Coulter, Brea, CA, USA) according to the manufacturer’s instructions and analyzed with the D-Code system (Bio-Rad, Hercules, CA, USA). After electrophoresis, the gel was stained with SYBER Gold (Molecular Probes, Eugene, OR, USA) and scanned with FLA-3000 (GE Healthcare Life Sciences, Marlborough, MA, USA). When PCR products from the soil DNA of the upper (0–2 cm) and lower (2–4 cm) parts were analyzed, the band patterns of DGGE gels were almost identical (data not shown). The DGGE analysis of the upper part soil indicated that the overall bacterial community structures were very similar among soils in P1–6 and AL1-6 including C1–4 and PF1–3 (Fig. 1). Although they were not major bands, at least two paddy field-specific bands, shown by arrow heads, were detected. Bacteria corresponding to bands 1 and 2 belonged to the phyla *Cyanobacteria* and *Acidobacteria*, respectively. Sequence identity between the two bands was 84%. Soils of the same land use contained very similar bacterial communities regardless of the altitude. This was consistent with the similar soil properties observed at different altitudes. The DGGE profiles of amplified 16S rRNA genes from the soils of natural slopes, including PF3, B1–3, G1, and CF1–2, were similar to each other (Fig. 1).

The pyrosequencing technique was considered to have high levels of robustness, consistency, and resolution (30). The hypervariable V4–V5 regions of the 16S rRNA gene were PCR-amplified using the primer pairs, F563-LXA and BSR926-LB (Table S3), with OneTaq DNA polymerase (New England Biolabs) from upper part (0–2 cm) soil. PCR products were purified by Agencourt AMPure XP with sizing buffer (7% PEG6000 and 1 M NaCl). Emulsion PCR was performed with the Lib-L kit (Roche, Branford, CT, USA), and amplicons were analyzed on the GS Junior 454 system (Roche). Raw sequences were processed and analyzed using QIIME (7) through OTUMAMi (23). After removing low quality sequences, the multiplexed reads were assigned to the corresponding soil samples based on their barcodes (9). Bacterial sequences were grouped into operational taxonomic units (OTUs) using a 97% identity threshold and taxonomically classified using the RDP naïve Bayesian rRNA Classifier (29).

A total of 132,997 optimized sequences and 15,258 OTUs were obtained from 20 soil samples of the 5 land use types (P, AL, C, PF, and CF). The heat map analysis at the genus level showed that the major bacterial community structures were almost similar in P1–6 (Fig. S1). This is consistent with the DGGE profiles. The same results were obtained from the heat map analysis of soil bacteria in AL1–6, C1–4, PF1–2, and CF1–2 (Fig. S1). An OTU belonging to *Acidobacteria* subdivision 6, which is the most phylogenetically diverse and numerous *Acidobacteria* subgroup, was the most abundant in all soils tested. The dominancy of an OTU belonging to the class *Anaerolineae* was greater in PF than in the other land use types. Since the major bacterial community structures were almost similar in each land use regardless of the altitude, we combined the OTU data of each land use from different altitudes. *Proteobacteria* and *Acidobacteria* were two major phyla in all the soils examined (Table 1). *Proteobacteria* was slightly more predominant in PF (Table 1). The relatively higher abundance of members of the phylum *Proteobacteria* in PF has recently been reported (14, 18). The phylum *Acidobacteria* was slightly more predominant in AL. The soil in AL was more acidic than that in P (Table S1). This may have been because of the reducing conditions in P. Although soil in P1–6 was not covered with water at the sampling time, reducing conditions may occur with irrigation, resulting in a higher pH. Previous findings showing that soil pH regulates the abundance of *Acidobacteria* (19, 22) support this phenomenon. Although its functional and ecological roles are poorly characterized, *Acidobacteria* may be adapted to an oligotrophic environment (8). The relatively lower total carbon content in AL (Table S1) may explain the abundance of *Acidobacteria*.

In order to determine differences in the relative diversity and richness of soil bacterial communities among land uses, the observed number of clusters, Chao1 richness, and Shannon diversity index were calculated at a genetic distance of 0.03. Bacterial species richness was slightly higher in PL than in the other land uses (Table 2). Bacterial diversity indicated by the Shannon diversity index was also slightly higher in PL than in the other land uses (Table 2). Several studies have documented stable bacterial diversity and richness in soil. A pristine forest and eight-year-old grassland surrounded by the same forest are previously shown to have the same bacterial diversity (25). Furthermore, soil bacterial diversity is found to be relatively unchanged among three types of soils: cropland, grassland, and forest (11). No marked differences are observed in microbial diversity levels among agricultural sites for more than 20 years with three different managements including tillage and the addition of extra nutrients and a non-agricultural grassland in Australia (4). Bacterial diversity is more dependent on soil pH than on land use types, such as primary and logged forests and crop and pasture lands, in the equatorial tropics of Southeast Asia (26). Since soil pH was basically acidic and not significantly different among the land use types in the present study (Table S1), the influence of pH on bacterial diversity appeared to be negligible.

No significant differences were noted in bacterial richness or diversity among soil samples; therefore, we attempted to establish whether bacterial community structures differed depending on land use. A beta-diversity analysis was performed using weighted UniFrac distance metrics. In order to assess differences, distances were used for a Principal Coordinate Analysis (PCoA) using QIIME (7) and visualized in 3D plots using Emperor with Jackknifing to estimate confidence intervals (28). The first three axes of PCoA accounted for 60% of the variation. Three well-defined clusters of land uses were observed (Fig. 2). These defined clusters were not observed in PCoA using soil physicochemical parameters (Fig S2), indicating that clustering cannot be explained by soil parameters only. The bacterial communities in CF, which are located on the natural slope, differed from those in the terraced P. Since the terraced P were constructed on the natural slope, the bacterial community structures had been changed from the natural-soil type to the paddy-field type. AL including C and PF contained different bacterial communities from those in P. When the lands were not used as P, the bacterial community structure changed to the AL type, but did not revert back to the natural-soil type. Although the periods of abandonment varied between 7 and 30 years, the AL analyzed in the present study showed the same bacterial community. Grassland resulting from deforestation for 8 years is previously shown to have the same bacterial community as that of a surrounding pristine forest (25). Even 30 years for abandonment is not sufficient for the bacterial community structure to revert back to the natural-soil type. Therefore, bacterial communities reflect the history of land use, from the natural slope to terraced P, then to AL.

We collected soils from terraced P before irrigation and did not analyze the soil bacterial community in flooded P. When bacterial communities in the bulk soil in Japanese P are analyzed from flooded to upland conditions, they are found to remain stable throughout the year (12). A previous study reports that the rotation from flooded P to non-flooded, un-used, or cultivated with maize does not significantly influence microbial community compositions in the Philippines (6). Based on these findings, we speculate that even under flooded conditions, the bacterial communities in P are similar to those before irrigation.

The sequenced read data have been deposited in the DDBJ Sequence Read Archive (DRA) under accession numbers DRA004024 and DRA004025.

## Supplementary Material









## Figures and Tables

**Fig. 1:**
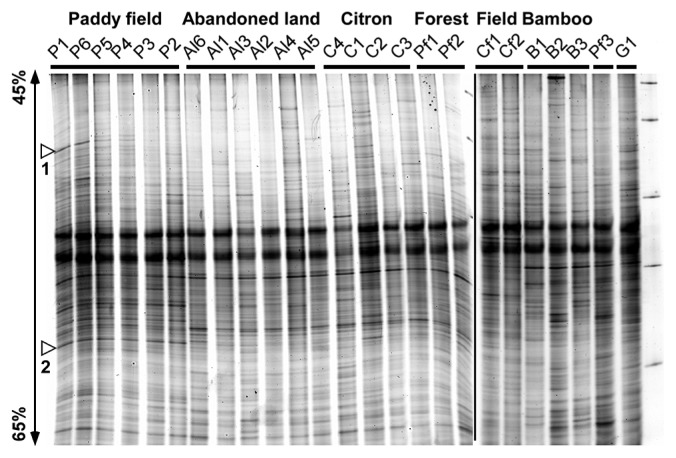
DGGE profiles of amplified 16S rDNA from soils of different land uses. Soil was collected using a 5-cm diameter plastic cylinder inserted into the soil with a hammer. The surface layer was removed in order to discard organic residues, plant roots, and shoots, and 2 samples at depths of 0–2 cm and 2–4 cm were collected from each individual sampling position. Soil samples were organized from lower to higher altitude locations for all land uses and analyzed on an 8% polyacrylamide gel with a 45–65% gradient of denaturant urea-formamide under a constant voltage of 50 V for 20 h. A detailed sample description of each land use: paddy fields (P), abandoned land (AL), citrus gardens (Citron, C), planted forests (Forest, PF), crop fields (Field, CF), bamboo thickets (Bamboo, B), and ginkgo (G), was shown in Table S1. Note that the dark bands observed in all lanes in the middle of the gels did not appear to be DNA because the relative position of these bands to the markers shifted between experiments.

**Fig. 2:**
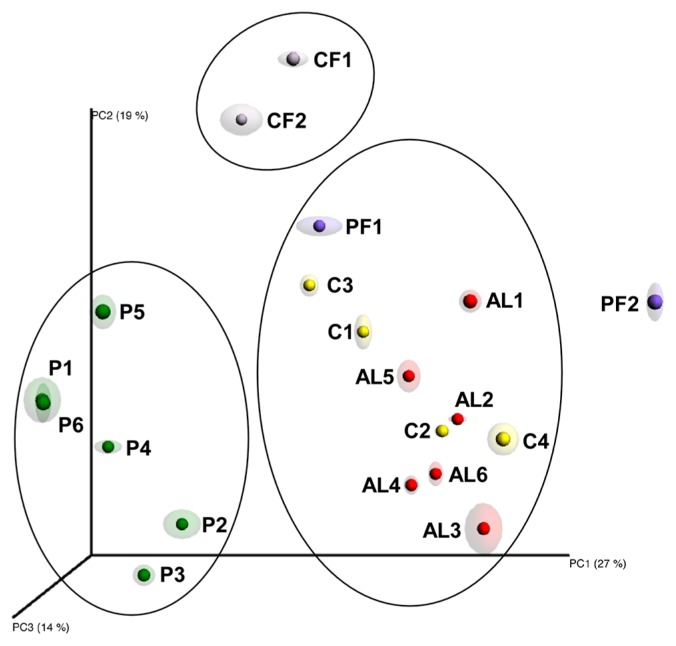
The Jackknifed Principal Coordinate analysis (PCoA) as a measure of β-diversity. The weighted distance metric between communities based on lineages was measured with weighted UniFrac. These metrics were used in three-dimensional PCoA plots. In order to visualize plot data, the visual tool software Emperor was used. Jackknifing estimated confidence by displaying ellipsoids around the samples. Different colors represent soil samples from different land use types. A description of soil samples was provided in Fig. 1.

**Table 1:** Abundant phyla in soil samples.

Phylum	Relative abundance^a^ (%)				

	Paddy fields	Abandoned land	Citrus gardens	Planted forests	Crop fields
***Acidobacteria***	**20 (4.1)**	**31 (6.7)**	**26 (3.3)**	**23 (5.9)**	**24 (6.8)**
*Actinobacteria*	3 (0.8)	2 (1.1)	3 (0.1)	3 (0.1)	5 (3.5)
*Bacteroidetes*	3 (2.8)	1 (0.5)	2 (1.7)	2 (0.2)	4 (2.5)
*Caldithrix*	1 (0.5)	1 (0.6)	1 (0.4)	1 (0.3)	1 (0.1)
*Chlorobi*	1 (0.2)	0	0	0	0
*Chloroflexi*	9 (2.6)	4 (0.5)	5 (0.8)	4 (2.4)	5 (1.8)
*Cyanobacteria*	2 (1.0)	0	1 (0.5)	0	1 (0.5)
*Firmicutes*	7 (3.7)	2 (1.2)	2 (0.5)	1 (0.8)	5 (2.1)
*Gemmatimonadetes*	2 (1.0)	3 (0.6)	2 (0.5)	2 (0.2)	3 (0.9)
*Nitrospirae*	2 (0.5)	4 (2.1)	2 (1.4)	4 (1.3)	2 (0.5)
OD1	0	1 (0.7)	1 (0.2)	0	0
OP11	1 (0.1)	0	0	0	0
*Planctomycetes*	3 (1.6)	4 (1.8)	4 (2.4)	5 (1.9)	2 (0.5)
***Proteobacteria***	**32 (2.3)**	**33 (4.2)**	**35 (3.4)**	**38 (5.5)**	**35 (0.8)**
*Spirochaetes*	1 (0.4)	0	0	0	0
*Unclassified bacteria*	1 (0.2)	1 (0.2)	1 (0.1)	1 (0.2)	1 (0.1)
*Verrucomicrobia*	7 (1.3)	10 (1.4)	11 (1.2)	12 (2.8)	10 (0.9)
WS1-6	1 (0.5)	2 (0.9)	2 (0.8)	2 (0.5)	1 (0.2)

a The average of multiple samples from different altitudes with the standard deviation (in parentheses). No significant difference was observed with Tukey-Kramer’s test at the 0.05 significance level.

**Table 2:** Richness and diversity of soil bacteria.

Land use	Sample/Asl. (m)		# of reads	Clusters^a^		Chao1^a^		Shannon diversity^a^ (H′)	
Paddy fields	P1	381	10311	2928	2193 (459)	4886	3848 (696)	7.06	7.03 (0.07)
	P6	389	5356	2131		3922		7.08	
	P5	461	3466	1576		2759		6.96	
	P4	530	6745	2274		3967		7.09	
	P3	602	4989	1890		3521		6.92	
	P2	655	7074	2363		4032		7.07	

Abandoned land	AL6	388	5399	1580	1933 (359)	2621	3249 (516)	6.59	6.77 (0.13)
	AL1	393	5075	1878		3267		6.90	
	AL3	449	8637	2341		3750		6.88	
	AL2	467	9831	2393		3808		6.85	
	AL5	606	4190	1494		2654		6.65	
	AL4	655	6546	1913		3394		6.77	

Citrus gardens	C4	398	8570	2265	1742 (352)	3872	3205 (458)	6.81	6.81 (0.07)
	C1	513	4249	1639		2913		6.86	
	C2	518	3799	1522		2898		6.72	
	C3	655	3627	1543		3137		6.86	

Planted forests	PF1	393	6890	2358	1944 (585)	4122	3424 (987)	7.10	6.63 (0.65)
	PF2	600	5096	1530		2726		6.17	

Crop fields	CF1	445	3483	1485	1800 (445)	2679	3167 (690)	6.87	6.88 (0.02)
	CF2	603	7796	2115		3654		6.90	

a The averages of multiple samples from different altitudes with the standard deviation (in parentheses) were indicated on the right. No significant difference was observed with Tukey-Kramer’s test at the 0.05 significance level.

## References

[1] Andrew D.R., Fitak R.R., Munguia-Vega A., Racolta A., Martinson V.G., Dontsova K. (2012). Abiotic factors shape microbial diversity in Sonoran desert soils. Appl Environ Microbiol.

[2] Balvanera P., Pfisterer A.B., Buchmann N., He J.S., Nakashizuka T., Raffaelli D., Schmid B. (2006). Quantifying the evidence for biodiversity effects on ecosystem functioning and services. Ecol Lett.

[3] Berthrong S.T., Buckley D.H., Drinkwater L.E. (2013). Agricultural management and labile carbon additions affect soil microbial community structure and interact with carbon and nitrogen cycling. Microb Ecol.

[4] Bissett A., Richardson A.E., Baker G., Thrall P.H. (2011). Longterm land use effects on soil microbial community structure and function. Appl Soil Ecol.

[5] Blasiak L.C., Schmidt A.W., Andriamiarinoro H. (2014). Bacterial communities in Malagasy soils with differing levels of disturbance affecting botanical diversity. PLoS One.

[6] Breidenbach B., Conrad R. (2015). Seasonal dynamics of bacterial and archaeal methanogenic communities in flooded rice fields and effect of drainage. Front Microbiol.

[7] Caporaso J.G., Kuczynski J., Stombaugh J. (2010). QIIME allows analysis of high-throughput community sequencing data. Nat Methods.

[8] Fierer N., Bradford M.A., Jackson R.B. (2007). Toward an ecological classification of soil bacteria. Ecology.

[9] Hamady M., Walker J.J., Harris J.K., Gold N.J., Knight R. (2008). Error-correcting barcoded primers for pyrosequencing hundreds of samples in multiplex. Nat Methods.

[10] Ikeda S., Watanabe K.N., Minamisawa K., Ytow N. (2004). Evaluation of soil DNA from arable land in Japan using a modified direct-extraction methods. Microbes Environ.

[11] Jangid K., Williams M.A., Franzluebbers A.J., Schmidt T.M., Coleman D.C., Whitman W.B. (2011). Land-use history has a stronger impact on soil microbial community composition than aboveground vegetation and soil properties. Soil Biol Biochem.

[12] Kikuchi H., Watanabe T., Jia Z., Kimura M., Asakawa S. (2007). Molecular analyses reveal stability of bacterial communities in bulk soil of a Japanese paddy field: Estimation by denaturing gradient gel electrophoresis of 16S rRNA genes amplified from DNA accompanied with RNA. Soil Sci Plant Nutr.

[13] Liliensiek A.K., Thakuria D., Clipson N. (2012). Influences of plant species composition, fertilization and *Lolium perenne* ingression on soil microbial community structure in three Irish grasslands. Microb Ecol.

[14] Lupatini M., Suleiman A.K.A., Jacques R.J.S., Antoniolli Z.I., Kuramae E.E., Camargo F.A.D., Roesch L. (2013). Soil-borne bacterial structure and diversity does not reflect community activity in Pampa biome. PLoS One.

[15] Mitchell R.J., Hester A.J., Campbell C.D., Chapman S.J., Cameron C.M., Hewison R.L., Potts J.M. (2010). Is vegetation composition or soil chemistry the best predictor of the soil microbial community?. Plant soil.

[16] Morimoto S., Ogawa N., Hasebe A., Fujii T. (2008). Isolation of effective 3-chlorobenzoate-degraders in soil using community analyses by PCR-DGGE. Microbes Environ.

[17] Mulvaney R.L., Sparks D.L. (1996). Nitrogen-inorganic forms. Methods of Soil Analysis. Part 3.

[18] Nacke H., Thürmer A., Wollherr A., Will C., Hodac L., Herold N., Daniel R. (2011). Pyrosequencing-based assessment of bacterial community structure along different management types in German forest and grassland soils. PLoS One.

[19] Naether A., Foesel B.U., Naegele V. (2012). Environmental factors affect acidobacterial communities below the subgroup level in grassland and forest soils. Appl Environ Microbiol.

[20] Navarrete A., Cannavan F.S., Taketani R.G., Tsai S.M. (2010). A molecular survey of the diversity of microbial communities in different Amazonian agricultural model systems. Diversity.

[21] Rodrigues J.L., Pellizari V.H., Mueller R. (2013). Conversion of the Amazon rainforest to agriculture results in biotic homogenization of soil bacterial communities. Proc Natl Acad Sci USA.

[22] Sait M., Davis K.E., Janssen P.H. (2006). Effect of pH on isolation and distribution of members of subdivision 1 of the phylum *Acidobacteria* occurring in soil. Appl Environ Microbiol.

[23] Satoh H., Oshima K., Suda W., Ranasinghe P., Li N., Gunawardana E.G.W., Hattori M., Mino T. (2013). Bacterial population dynamics in a laboratory activated sludge reactor monitored by pyrosequencing of 16S rRNA. Microbes Environ.

[24] Singh B.K., Millard P., Whiteley A.S., Murrell J.C. (2004). Unravelling rhizosphere-microbial interactions: opportunities and limitations. Trend Microbiol.

[25] Suleiman A.K., Manoeli L., Boldo J.T., Pereira M.G., Roesch L.F. (2013). Shifts in soil bacterial community after eight years of land-use change. Syst Appl Microbiol.

[26] Tripathi B.M., Kim M., Singh D., Lee-Cruz L., Lai-Hoe A., Ainuddin A.N., Adams J.M. (2012). Tropical soil bacterial communities in Malaysia: pH dominates in the equatorial tropics too. Microb Ecol.

[27] Truog E. (1930). The determination of readily available phosphorus of soils. J Am Soc Agron.

[28] Vázquez-Baeza Y., Pirrung M., Gonzalez A., Knight R. (2013). EMPeror: a tool for visualizing high-throughput microbial community data. Gigascience.

[29] Wang Q., Garrity G.M., Tiedje J.M., Cole J.R. (2007). Naïve Bayesian classifier for rapid assignment of rRNA sequences into the new bacterial taxonomy. Appl Environ Microbiol.

[30] Winsley T., van Dorst J.M., Brown M.V., Ferrari B.C. (2012). Capturing greater 16S rRNA gene sequence diversity within the domain Bacteria. Appl Environ Microbiol.

[31] Wu T., Chellemi D.O., Graham J.H., Martin K.J., Rosskopf E.N. (2008). Comparison of soil bacterial communities under diverse agricultural land management and crop production practices. Microb Ecol.

