# Supplementary material for: Effects of Land Use Changes from Paddy Fields on Soil Bacterial Communities in a Hilly and Mountainous Area
Source: Microbes Environ. 2016 Apr 19;31(2):160–4. doi: 10.1264/jsme2.ME15187 (PMC4912151; doi:10.1264/jsme2.ME15187)
Supplement: Supplementary file 1 [file 31_160_s1.pdf]

Rokunuzzaman et al. Figure S1

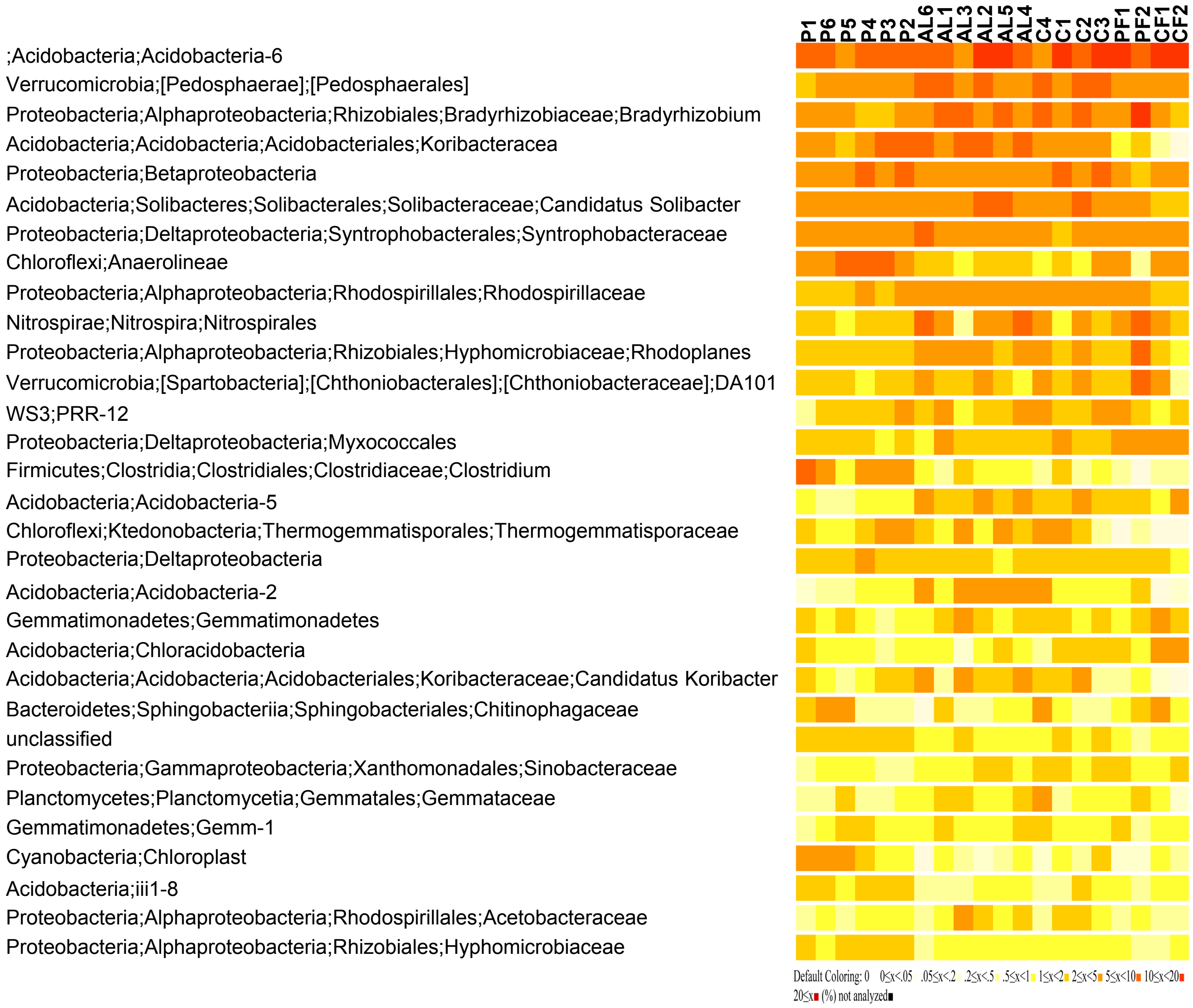

Fig. S1. The heat map analysis at genus level. The heat map was constructed with OTUMAMi using the taxonomic information from OTUs. Description of the soil samples has been provided in Fig. 1.

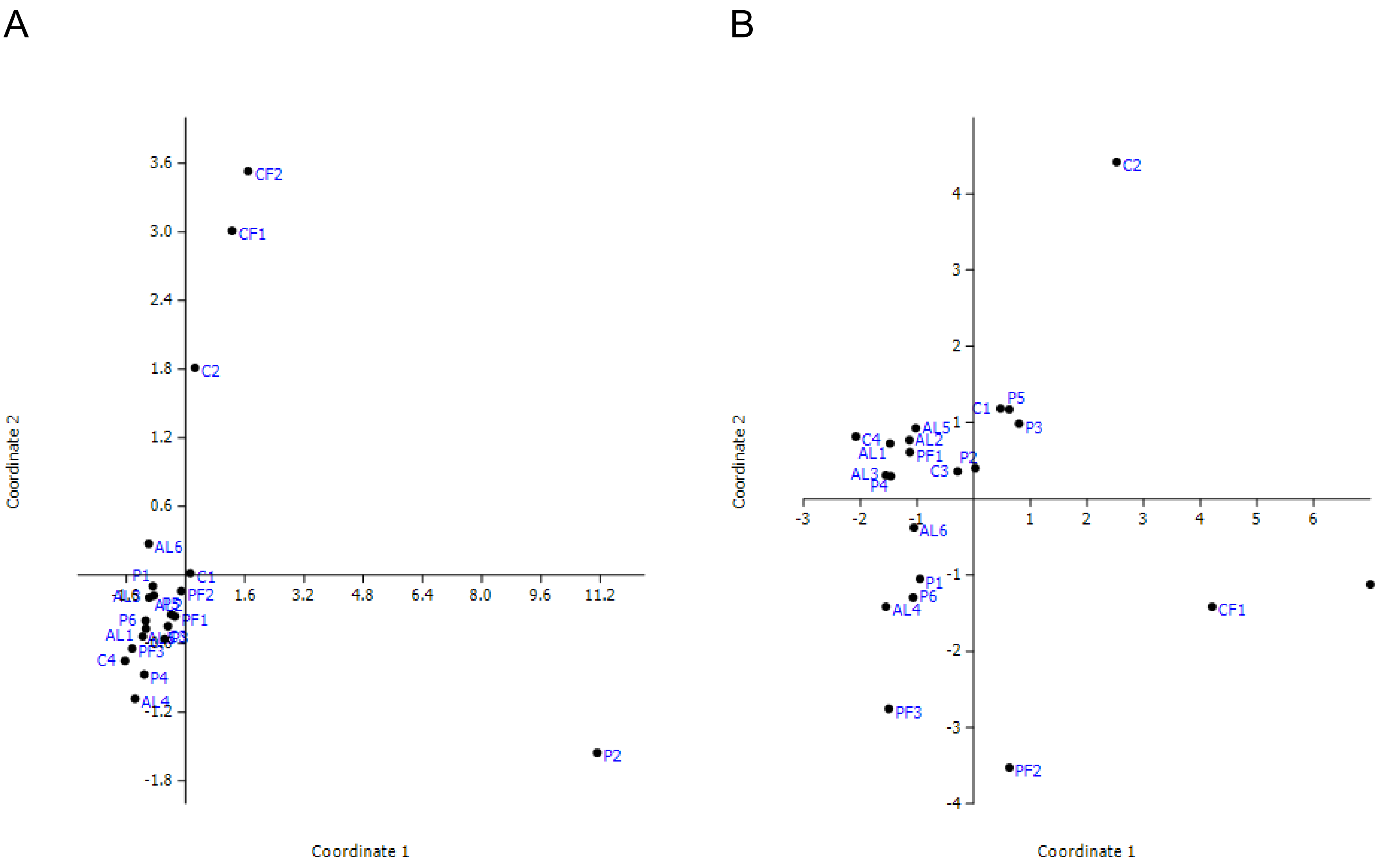

Fig. S2. The Principal Coordinate analysis using soil parameters. The Principal coordinates were analyzed and visualized with software package PAST v3.10 using (A) all chemical parameters, (B) all physicochemical parameters, (C) pH and total carbon, and (D) pH, total carbon and total nitrogen.

C

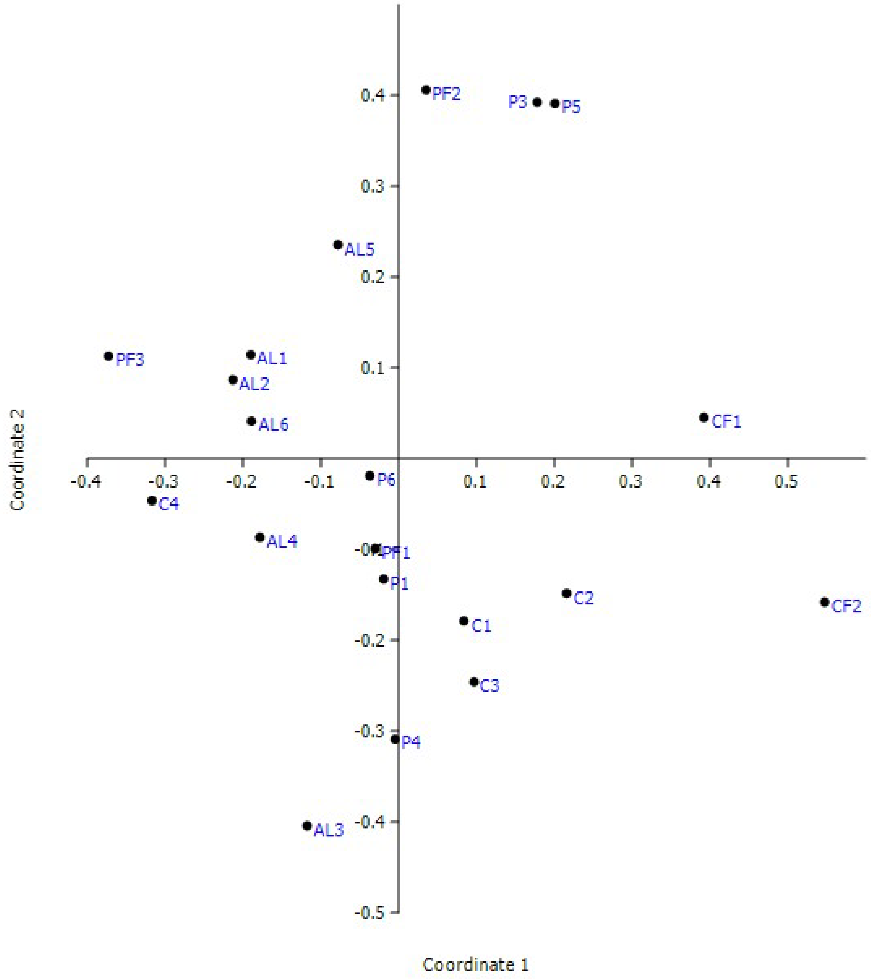
PF2
P3
P5
AL5
PF3
AL1
AL2
AL6
CF1
P6
C4
AL4
PF1
P1
C2
CF2
C1
C3
P4
AL3
Coordinate 1
Coordinate 2

D

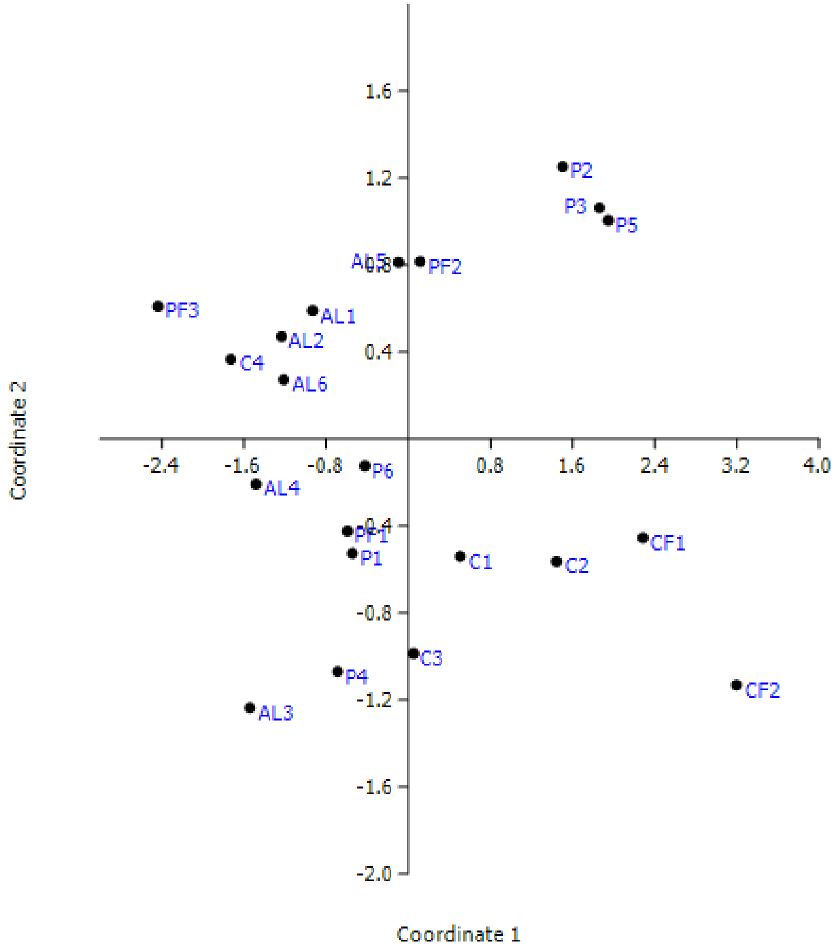
P2
P3
P5
AL5
PF2
PF3
AL1
AL2
C4
AL6
P6
AL4
PF1
P1
C1
C2
CF1
C3
P4
AL3
CF2
Coordinate 1
Coordinate 2
