# Supplementary material for: Effects of Land Use Changes from Paddy Fields on Soil Bacterial Communities in a Hilly and Mountainous Area
Source: Microbes Environ. 2016 Apr 19;31(2):160–4. doi: 10.1264/jsme2.ME15187 (PMC4912151; doi:10.1264/jsme2.ME15187)
Supplement: Supplementary file 4 [file 31_160_s4.doc]

Table S3. primers used in this study

| name | sequence (5′ to 3′) |
| --- | --- |
| for DGGE |  |
| 341F-GC | cgcccgccgcgcgcggcgggcggggcgggggcacggggggCCTACGGGAGGCAGCAG |
| 534R | ATTACCGCGGCTGCTGG |
| for pyrosequencing | |
| F563-L1A | CCATCTCATCCCTGCGTGTCTCCGACTCAG**ACGAGTGCGT**AYTGGGYDTAAAGNG |
| F563-L2A | CCATCTCATCCCTGCGTGTCTCCGACTCAG**ACGCTCGACA**AYTGGGYDTAAAGNG |
| F563-L3A | CCATCTCATCCCTGCGTGTCTCCGACTCAG**AGACGCACTC**AYTGGGYDTAAAGNG |
| F563-L4A | CCATCTCATCCCTGCGTGTCTCCGACTCAG**AGCACTGTAG**AYTGGGYDTAAAGNG |
| F563-L5A | CCATCTCATCCCTGCGTGTCTCCGACTCAG**ATCAGACACG**AYTGGGYDTAAAGNG |
| F563-L6A | CCATCTCATCCCTGCGTGTCTCCGACTCAG**ATATCGCGAG**AYTGGGYDTAAAGNG |
| F563-L7A | CCATCTCATCCCTGCGTGTCTCCGACTCAG**CGTGTCTCTA**AYTGGGYDTAAAGNG |
| F563-L8A | CCATCTCATCCCTGCGTGTCTCCGACTCAG**CTCGCGTGTC**AYTGGGYDTAAAGNG |
| F563-L9A | CCATCTCATCCCTGCGTGTCTCCGACTCAG**TAGTATCAGC**AYTGGGYDTAAAGNG |
| F563-L10A | CCATCTCATCCCTGCGTGTCTCCGACTCAG**TCTCTATGCG**AYTGGGYDTAAAGNG |
| BSR926-LB | CCTATCCCCTGTGTGCCTTGGCAGTCTCAGCCGTCAATTYYTTTRAGTTT |

A 40-bp GC clamp was indicated with small letters. Bold sequences indicate the barcoded tags.
